# Ovariotomy during Pregnancy

**Published:** 1877-12

**Authors:** 


### OVARIOTOMY DURING PREGNANCY.

In closing the discussion of the subject, Mr. Wells said he had, according to his memory, declined to operate upon eight or nine patients who had applied to him for relief from this disease—about as many as he had operated upon; and some had declined to accept the operation after he had recommended it. No rule could be laid down to direct the action of a member of the profession as to his course in a given case; the case must declare the remedy. Such questions as the general health and surroundings of the patient, age, the puerperal state, and the rapidity of growth in the tumor, would come up for consideration, and must be decided by the judgment and experience of the surgeon at the time. With many patients the only hope of escape from death was through the operation, and the success of the operation in his hands in the past would, should occasion require, warrant him in recommending it in the future.—*American Practitioner.*

